# Medication safety in acute care in Australia: where are we now? Part 2: a review of strategies and activities for improving medication safety 2002-2008

**DOI:** 10.1186/1743-8462-6-24

**Published:** 2009-09-22

**Authors:** Susan J Semple, Elizabeth E Roughead

**Affiliations:** 1 Quality Use of Medicines and Pharmacy Research Centre, Sansom Institute, University of South Australia, GPO Box 2471, Adelaide, 5001, Australia

## Abstract

**Background:**

This paper presents Part 2 of a literature review examining medication safety in the Australian acute care setting. This review was undertaken for the Australian Commission on Safety and Quality in Health Care, updating the 2002 national report on medication safety. Part 2 of the review examined the Australian evidence base for approaches to build safer medication systems in acute care.

**Methods:**

A literature search was conducted to identify Australian studies and programs published from 2002 to 2008 which examined strategies and activities for improving medication safety in acute care.

**Results and conclusion:**

Since 2002 there has been significant progress in strategies to improve prescription writing in hospitals with the introduction of a National Inpatient Medication Chart. There are also systems in place to ensure a nationally coordinated approach to the ongoing optimisation of the chart. Progress has been made with Australian research examining the implementation of computerised prescribing systems with clinical decision support. These studies have highlighted barriers and facilitators to the introduction of such systems that can inform wider implementation. However, Australian studies assessing outcomes of this strategy on medication incidents or patient outcomes are still lacking. In studies assessing education for reducing medication errors, academic detailing has been demonstrated to reduce errors in prescriptions for Schedule 8 medicines and a program was shown to be effective in reducing error prone prescribing abbreviations. Published studies continue to support the role of clinical pharmacist services in improving medication safety. Studies on strategies to improve communication between different care settings, such as liaison pharmacist services, have focussed on implementation issues now that funding is available for community-based services. Double checking versus single-checking by nurses and patient self-administration in hospital has been assessed in small studies. No new studies were located assessing the impact of individual patient medication supply, adverse drug event alerts or bar coding. There is still limited research assessing the impact of an integrated systems approach on medication safety in Australian acute care.

## Background

This paper is the second in a two-part literature review of medication safety in the Australian acute care setting [1]. It examines the Australian evidence-base for strategies to reduce medication errors and updates the data presented in the *Second National Report on Patient Safety - Improving Medication Safety *[2].

International evidence has identified a number of tools or practices that may reduce medication incidents in healthcare. Computerised physician order entry (CPOE) coupled with clinical decision support systems (CDSS) is amongst the most studied. These systems allow standardised online prescribing coupled to computerised advice to support prescribing decisions. The systems may provide automated checks or alerts such as drug-drug interaction checking or drug allergy alerts. These systems can reduce adverse drug events in hospital settings [3] although there have been reports of increased error rates when implementation problems have occurred [4]. Most studies have examined "home-grown" CPOE/CDSS systems developed and used within an institution; commercially developed systems have been less well studied [3].

International studies also support the role of clinical pharmacists in hospital wards for preventing adverse drug events [4-6]. Other strategies examined for improving medication safety in acute care include individual patient supply of medication, robotic systems for medication dispensing (including automated bedside systems), bar coding of medication packaging and patients, intravenous devices capable of performing dilutions, computerised medication records, changes to systems to facilitate improved communication and educational interventions [4,7,8].

This review examines the published literature on the evidence and implementation of these strategies in the Australian acute care setting. While these approaches may impact particular stages in the medication use process (such as prescribing, transcribing of information, dispensing, administration), no single strategy can target all stages of the complex process. While the evidence for these strategies has been reviewed separately the importance of using a multifaceted approach, incorporating a number of these strategies, is emphasised to improve medication safety.

The analysis of medication incident reports occurring within an institution can allow problems with systems to be identified and corrected. "Root cause analysis" is one technique which has been adapted from industries such as aviation and aerospace for use in the health care setting [9]. This involves identifying the "root" and contributory factors that have led to a medication incident rather than seeking to blame the individual/s involved. This information can be used to formulate strategies to prevent similar incidents, which in turn can be evaluated as part of a continuous quality improvement cycle [10]. In this review, root cause analysis strategies were included, as were systems to improve the reporting of medication incidents in acute care.

## Methods

### Search strategy

Databases and search terms used in the search strategy have been detailed in Part 1 of the review [1]. Criteria relevant to the general headings in the *Second National Report on Patient Safety - Improving Medication Safety *[2] were used.

The database search was supplemented with review of relevant reports and resources on the Australian Commission on Safety and Quality in Health Care website . Additionally, the website of the Australian Government Department Health and Ageing was searched for information and publications relating to government electronic health initiatives including bar coding, shared electronic medical records and electronic prescribing. State government websites and the website of the New South Wales Therapeutic Advisory Group (NSW TAG) were searched for information on medication safety initiatives in hospitals.

### Selection of studies for review

Letters, study reports and reviews published between 2002 and 2008 were selected if they reported on:

- systems to promote improved prescription writing;

- systems to promote dissemination of information about medicines and improved prescriber decision-making;

- systems used to promote accurate dispensing and/or distribution of medicines;

- systems to ensure adequate checking;

- systems used to promote accurate administration of medicines;

- systems to improve management of medicines;

- medicine-specific handling/management strategies;

- clinical pharmacy services;

- systems to improve information transfer about medicines between hospital and community settings;

- medication management services and case conferencing;

- systems-based approaches to understanding and preventing medication errors;

- systems to promote reporting of medication incidents and adverse drug reactions in hospitals.

Studies undertaken in the community setting were excluded.

## Results and Discussion

### Evidence for systems to improve prescription writing

#### The National Inpatient Medication Chart

A National Inpatient Medication Chart (NIMC) aims to improve medication safety through standardisation of medication ordering in all Australian public hospitals, and a number of private hospitals. The NIMC was adopted for national roll-out following an agreement of the Australian Health Ministers Council in 2004 [11]. The recommendation for a process of pharmaceutical review of all aspects of medication management in the hospital was also part of this reform [12].

The chart along with education on safe prescribing and administration were piloted in 31 public and private hospitals in metropolitan, regional and rural areas in 2004 [13]. Pre and post-implementation audits were undertaken by 28 sites. Improvements seen in the combined results included:

- increased documentation of adverse drug reactions (21% at baseline to 50% at three months);

- decreased prescription of drugs to which patient had an allergy (9% to 6%);

- increased entry of actual administration times (18% to 68%);

- increased frequency of providing the indication for a 'prn' (as needed) medication (13% to 26%);

- increased documentation of the maximum dose for a 'prn' medication (24% to 36%);

- increased frequency of the prescriber name being identifiable (41% to 79%); and

- increased frequency of target international normalised ratio (INR) documentation (9% to 71%) [13].

The study used surrogate, rather than direct, measures of patient harm [13].

An implementation strategy for the NIMC in three Victorian hospitals involved an interdisciplinary steering group to support integration across the whole organization, a dedicated project officer acting as facilitator and coordinator and four interdisciplinary working groups addressing supply, communication, education and evaluation issues [14].

An audit of the NIMC design and performance was also undertaken at the Royal Perth Hospital (RPH) [15]. Chart design assessment was performed on 15 design features and compared with the previously used RPH chart, four other WA hospital charts and nine charts from teaching hospitals in other states and territories. Completion of the individual fields of the chart was assessed and an audit of NIMC charts from six medical and surgical wards of the hospital was compared to completion of the previous RPH chart. Aspects of the design of the NICM considered likely to improve medication safety included a) a section to complete medication history; b) provision for recording sustained-release dose forms; c) a mechanism to circle inpatient drugs intended for provision at discharge; d) provision for documenting a medicine's indication; and e) direction to record intended administration times of each medicine. Four of these five advantages were poorly complied with in practice. Overall compliance with the NIMC was 56% [95% CI 43-67%]. Only 2.3% of charts had the medication history completed. There was a modest, but non-significant increase in overall compliance after the introduction of the NIMC compared to the previous chart (6% [95% CI -0.2 to 13%]). Concerns about design features of the chart included cramped design, lack of colour, lack of provision for variable dosing and the need for, on average, twice as many charts per admission (and hence increased requirements for rewriting charts and possible transcription errors) [15].

Tools have been developed to allow ongoing evaluation of the chart [16]. Currently local management of the NIMC is overseen by local jurisdictional bodies at the State and Territory level [17]. Where relevant, the local body will refer proposed changes to the national NIMC Oversight Committee. Versions of the NIMC for paediatric patients and patients requiring long hospital stays are available [18,19].

#### Prescriber education

Education services to individual healthcare professionals are sometimes referred to as 'academic detailing'. This term refers to an educational approach based on principles of communications theory and behaviour change [20].

A 2001 NSW study examined whether academic detailing could reduce prescription errors for drugs of addiction (DOA) in the hospital setting [21]. Errors assessed included:

- the quantity not written in both words and numbers;

- the DOA not written on a separate script;

- alterations to the script not initialled;

- details of the preparation or form of the drug omitted;

- liquid preparations without a milligram dose given;

- strength omitted or incorrect.

The intervention involved a one-on-one academic detailing session for all first and second year post-graduate practitioners. A follow-up session was conducted after two months. Error rates were assessed two months after the intervention and compared with a control hospital. The baseline levels of errors at the intervention hospital (approximately 40%) were higher than those of the control hospital (25%), making comparison difficult. Error rates reduced from 41% of 46 prescriptions pre-intervention to 24% of 128 prescriptions post-intervention (p < 0.001, chi square = 17.3). There was no change in the error rate at the control hospital. No assessment was made on the likely to impact of these errors on patient outcomes.

Further adequately controlled studies are required to confirm whether academic detailing can reduce prescription error rates in the Australian hospital setting. These should include assessment of errors likely to impact on patient outcomes.

A pre-intervention, post-intervention study in a Melbourne teaching hospital examined an educational intervention to reduce the use of "error-prone prescribing abbreviations" in an emergency department (ED) [22]. The intervention, involving ED registrars and postgraduate course nurses, included small group and one-to-one tutorials about abbreviations commonly causing medication errors or confusion. Summary cards and posters reinforced this information. The intervention ran for six-months. All medication and fluid charts in the ED department were assessed for error-prone abbreviations at a randomly selected time each day for one week before the intervention and one week following. Abbreviations were classified as being of major, moderate or minor significance by two independent pharmacists. Charts for 166 patients were included in the two assessment phases. The error-prone abbreviation rate per 100 prescriptions decreased from 31.8 pre-intervention to 18.7 post-intervention (p < 0.001). The rates of abbreviations classified as of major significance decreased from 5.8 per 100 prescriptions pre-intervention to 2.3 post-intervention (p < 0.001). The study was not controlled and the sustainability of the effect over time is unknown.

### The evidence for systems ensuring better dissemination of knowledge about drugs

#### Clinical decision support systems (CDSS) with computerised physician order entry (CPOE)

In the 2002 national patient safety report, careful implementation and evaluation of electronic prescribing with decision support was suggested as a priority for improving medication safety in Australia [2]. Implementation research has since been conducted in Australia, however, outcome studies are still lacking.

The limitations of using electronic prescribing alone are highlighted in the findings of a 2001 study of discharge prescriptions at a teaching hospital in Brisbane [23]. A computer-generated discharge summary system enabled a discharge prescription to be generated based on information entered by the medical officer. An observational audit of 200 discharge prescriptions was conducted; involving 100 handwritten prescriptions (605 medications) and 100 computer generated prescriptions (700 medications). The same medical staff were responsible for both prescription types. There were more errors in the computer generated prescriptions (81 errors, 11.6% of items) compared to the handwritten ones (30 errors, 5.0% of items, p < 0.001). Errors judged to have the potential to result in patient harm were similar between the groups. Errors that occurred more frequently when a computer was used included dosing errors (25 computer generated compared with 5 handwritten errors) and duration of therapy errors due to default settings in the computer. The authors concluded that electronic prescribing without decision support and alerting systems could increase the risk of patient harm.

Recognition that electronic medication management systems can introduce machine-related errors led a University of NSW research group to develop a multilevel "accident model" to examine points in electronic prescribing systems where system failures may occur [24]. This model used a systematic approach to identify human-computer interaction processes as well as the context in which electronic prescribing systems are used (such as health professional cultures, organizational factors). The aim is to aid the development of electronic prescribing systems with features that improve patient safety. The validity of the model is still to be tested.

Implementation of an inpatient electronic prescribing and clinical decision support system in an acute and sub-acute ward at a metropolitan and rural hospital in Victoria found mixed results [25]. The system allowed a 'point and click' method of prescribing for physicians' ordering inpatient and discharge medications, integration with the hospital's pharmacy ordering system and use of clinical decision support tool. The clinical decision support allowed checking of interactions, allergies and duplicate ordering and access to drug information including the AusDI drug and therapeutics database and MIMS. The system was tested in one acute and one sub-acute 30-bed ward within each hospital. In each ward two clinical computers were available, in addition to a wireless laptop as a point-of-care computer. Intensive training was provided to medical officers and nursing staff. Use of the system in the acute ward setting was discontinued after six weeks in the rural hospital and eight weeks in the metropolitan hospital. The barriers to effective implementation in the acute ward setting were perceptions of increased clinical risk, workload issues, lack of medical staff commitment, insufficient computer access and technical and software limitations (including inadequate interaction and allergy checking, and problems with version control). The experience in the sub-acute ward setting in both hospitals was different, with use becoming accepted practice and the system being rolled out to other sub-acute wards. Medication error rates were not assessed. The study highlighted the need for document control, understanding clinical workflow issues and commitment through the whole organization. One of the barriers in the acute care setting was the need to frequently review medication charts and a ban on handwritten alterations to charts. The authors concluded that implementation of electronic prescribing and clinical decision support systems requires a highly organised approach at all levels of the institution, giving consideration to the technical and cultural issues and environment in which it is to be used.

Another qualitative feasibility study for an 'electronic prescribing decision support' (EPDS) system was undertaken in a NSW public hospital [26]. The Sauer's Triangle of Dependencies model was used to examine the organisational context in which an information system is placed. The study used interviews and focus groups with medical staff, pharmacists, nurse managers and clinical information technology experts. Questions examined the limitations of the present paper-based prescribing system, technical requirements for an electronic prescribing and decision support system, the environment in which the system would be used, the political setting, the type of ward structure suited to electronic prescribing, perceived barriers to implementation and mechanisms for consulting with medical staff in the design and implementation of an information system. Only 9% of medical staff and 20% of nurse managers participated. Results found that while nearly all participating clinicians indicated they would be willing to adopt electronic prescribing and alert systems, the implementation would be hampered by existing barriers. Barriers included lack of confidence in the security aspects of the system (e.g. use of electronic signatures, failing to log-out of a system), lack of funding and resources, concern about the time taken to complete and check an electronic prescription (e.g. excessive drug-drug interaction checking by some decision support/alerting systems), lack of compatibility with the existing patient administration system in the hospital and legislative barriers (such as legal requirement for handwritten signatures). The majority of clinicians favoured the idea of a state-wide EPDS system. Clinicians stated the need for an integrated system that would overcome the need to log into different systems for different types of results.

A 2007 project reviewed electronic medication management (EMM) systems in the Australian setting [27]. A multidisciplinary reference group including medical, pharmacy and nursing representation as well as clinical information technology experts formulated "key principles and core features" for assessing the suitability of an EMM systems. Systems from 11 companies were evaluated with all found to have the majority of the required core functions seen as important by the reference group. Systems were not ranked relative to each other. It concluded that a number of available systems were suitable for use in Australian hospitals, but that change management issues needed to be addressed for implementation to occur more widely.

The Australian Health Information Council (AHIC) provides advice to the Australian Health Ministers Advisory Council (AHMAC) on information management and technology in the health care system. A report by the AHIC in 2008 [28] examined Electronic Decision Support systems, with a focus on medicines, including the current state of their implementation in Australia. This review found there were some sound evidence-based programs used in hospitals in some States. In Victoria, for example, the Victorian Clinical Systems provided through Health *SMART *supported electronic prescribing and decision-making by providing patient allergies, adverse reactions and automatic checking for duplication and drug interactions. However, the report highlighted that an agreed set of national standards was lacking. The need to work with professional bodies to examine possible barriers and incentives for healthcare professionals in the uptake of electronic decision support was identified, as was the need for the health care workforce to be adequately supported to gain the skills to use the technology [28].

Since the previous medication safety review [2], a number of new studies have examined the implementation of electronic prescribing in combination with clinical decision support systems in Australian hospitals, but not the impact on medication error or patient outcomes. The potential value of these strategies now appears to be more widely recognised by government, hospitals and health professionals. Published studies provide useful insights into some of the barriers to the introduction of this strategy in the acute care setting that need to be considered with wider implementation.

### Evidence for systems promoting better medication distribution

#### Individual patient based medication distribution

In the 2002 review of medication safety in Australia [2], individual patient medication supply systems were found to reduce medication errors. Two Australian studies had compared different medication distribution methods and resultant errors associated with administration in the Australian hospital setting [29,30], showing a decrease in error rates. No further Australian studies were located. The Society for Hospital Pharmacists of Australia Standards of Practice for the Distribution of Medicines in Australian Hospitals published in 2006 [31] state that unit-dose systems are the preferred method of medicines distribution for healthcare facilities in terms of patient safety. However, there is a lack of published data on the uptake of individual patient supply systems for medications in Australian hospitals despite evidence supporting its use in reducing medication errors.

#### Automated dispensing devices

Automated medication dispensing devices are electronic storage devices that dispense medications in a controlled manner and track the use of medication. The 2002 medication safety review [2] noted that evidence for automated drug distribution systems in reducing medication incidents was limited. Two studies that evaluated automated drug distribution in the Australian health care setting [32,33] did not provide clear evidence of the efficacy of automated systems for reducing error. No further Australian studies since 2002 were located.

### Evidence for systems ensuring adequate checking

#### Bar coding

In the previous review [2] there was international evidence to support the investigation of bar coding as a strategy to reduce medication error. Bar-coding or other identification systems such as radio frequency identification tags could allow medication packaging supplied for an individual patient to be cross-checked with patient identification information (such as a hospital patient identification bracelet) at the point of medication administration [34]. Alternatively, more advanced systems could allow electronic prescriptions to interface automated dispensing systems to assemble individual medication packs and track administration [34]. No published studies in the Australian setting were identified.

An ongoing project administered by GS1 Australia (formerly EAN Australia) and the National E-Health Transition Authority (NEHTA) aims to implement an Australian standard coding system for medicines - the Australian Catalogue of Medicines (ACOM) [35]. This will ensure that all prescription and non-prescription medicines (including complementary medicines) have a globally unique code. A national coding system is required to allow the electronic transmission, storage and use of medication information. This has the potential to facilitate the use of bar coding technology.

#### Computer adverse drug event detection and alerts

A computer system may be used to detect potential adverse drug events (such as interactions between different medications, or abnormal laboratory results for a patient taking a particular medication) and alert a patient's health care professional such as physician or pharmacist. The previous review [2] found limited international evidence but a lack of Australian research to support the use of such systems for improving medication safety. No more recent Australian studies were located.

#### Single-person versus double-person checking by nurses administering medications

A study in a Victorian acute care hospital examined the safety of single-checking by a registered nurse of medications that had required double-person checking [36]. These included medications requiring calculations, drugs of addiction, cytotoxics, new drugs, epidurally administered drugs, variable dose insulin, blood products and high dose potassium chloride. Medication incident reports were assessed in the single checking study for a seven-month period and compared to those in the same units in the same months of the previous year when double-person checking was standard practice. There was no significant difference between the two periods, however the number of reported administration errors was low (four in the study period and five in the previous year), and the required study power to detect a difference was not reported. This study analysed medication incidents reported through the hospital's reporting scheme and did not include any independent assessment of errors. Reliance on incident reports may have meant errors were undetected as incidents are known to be under-reported. Further studies are required to provide conclusive evidence about the relative safety of single and double-person checking of high risk medications in the Australian acute care setting.

### Evidence for systems to improve medication administration

#### Drug packaging, storage and administration equipment

The potential for administration of IV medications by the wrong route has been highlighted by cases of inadvertent spinal administration of the anti-cancer medication vincristine [37]. The Society for Hospital Pharmacists of Australia has recommended strategies to reduce the risk of error associated with cytotoxic medications, with the abolition of syringes for administration of vincristine in favour of an infusion bag [37].

A system to prevent infusions being administered by the incorrect route has been developed at the Women's and Children's Hospital, Adelaide [38]. The system, called the Adelaide Regional Connector (ARC), was under prototype development in 2002. This colour coded luer incompatible system ensures that syringes and other drug administration equipment used to administer epidural and intrathecal doses were not able to be connected to those used to administer IV infusions.

Further research is needed to examine the current state of implementation of system changes to reduce the risk of inadvertent administration of IV and intrathecal medications by the wrong route.

Incorrect IV administration of potassium chloride can potentially cause significant patient harm [39]. The Medication Safety Taskforce of the previous Safety and Quality Council recommended components to be included in guidelines for potassium chloride [40] and case studies from two Australian hospitals were developed and made available online.

A root cause analysis of an incident in which a bolus dose of IV potassium chloride was inadvertently administered at the Alfred hospital in Melbourne [41] led to the development of pre-mixed solutions. These were developed by physician consensus and in collaboration with the product manufacturer. This allowed all concentrated potassium chloride preparations to be removed from all general wards. A policy for prescribing potassium chloride in millimoles rather than grams was also implemented. Outcomes were not evaluated.

#### Patient self-administration in the acute care setting

A pilot study in a Nursing Convalescent Unit of a large metropolitan teaching hospital examined the effectiveness of an inpatient self-medication program [42]. The six-month study examined three levels of administration: 1) registered nurse (RN) administration; 2) patient medication with direct supervision from an RN; and 3) self-medication with indirect RN supervision. Patient education and a medication record card were key components of the program. A total of 220 patients participated, with 45% remaining on Level 1, 26% reaching level 2 and 29% reaching level 3. There were no patient initiated medication errors in the study period and two errors involving staff, compared to one error in the previous six-month period. This study was conducted in a specialized unit. The findings, therefore, are not generalisable to other acute care settings but warrant further investigation.

#### Education and training about medication administration errors

An orientation program for newly employed registered nurses at a Queensland teaching hospital examined the ability of nurses to identify medication errors and apply strategies to prevent medication incidents [43]. The program used simulated medication administration scenarios of frequently occurring medication errors with potential for harm. After each scenario the nurses were asked whether they detected the errors, whether they would have modified their practice and whether they were aware of the error concept. Nurses were presented with education about concepts of human error and risks, the systems in place in the hospital to prevent medication errors, roles and responsibilities in detecting errors and preventing harm. The study was conducted over a two-year period with 591 nurses participating. Results showed that the risk would have been identified and appropriate action taken in a median of 5 and average of 4 of the 6 scenarios. This study did not assess whether this translated into improved recognition of medication errors in practice. Further research on the impact of education on incident rates and medication error detection in the acute care setting is still needed.

### Evidence for systems providing clinical pharmacy services

In the previous review [2] some Australian studies were located which supported the role of clinical pharmacists in improving patient safety. Newer studies, published since 2002, further support this role.

A pre-test, post-test study examined the impact of an emergency department (ED) clinical pharmacist on prescribing errors in a Victorian metropolitan teaching hospital [44]. Prescription error rates for patients during a 5 day control period were compared with error rates in the following week when a pharmacist ED service was provided. In the intervention period a dedicated ED pharmacist interviewed patients using a structured medication reconciliation form to obtain a medication history and reconciled the history with the ED medication chart where possible or passed the information to the ward pharmacist. At 24 hours post-admission a senior clinical pharmacist reviewed the medication history and medication chart and recorded and resolved any prescribing errors. Error types were classified using an in-house classification system and the risk rating was assessed by a blinded, independent physician using standard risk assessment criteria. There were 56 patients in the control period and 55 in the intervention period with patient characteristics and the number of drugs ordered per patient similar between groups. There were 88 prescription errors detected in the control period (1.6 errors/patient) and 25 in the intervention period (0.5/patient) (p < 0.0001). There was a relative reduction of errors rated as high/extreme (64% reduction), moderate (71% reduction) and minor (90% reduction). This study supports the role of an ED pharmacist in reducing prescription errors, however further controlled studies in other acute care hospitals in Australia are needed to determine the generalisability of these findings.

Less rigorous evidence for the effectiveness of clinical pharmacy interventions is provided by studies in which interventions undertaken by clinical pharmacists have been independently reviewed in order to assess their clinical significance or impact on patient outcomes or medication error rates. A number of studies of this type have been undertaken in the Australian setting as summarised in the previous review [2]. The largest study conducted in Australian acute care was published in 2004 [45]. Clinical pharmacist interventions in eight major public hospitals over an average of 21 days were reviewed by an independent multidisciplinary panel. There were 1,399 interventions during 24,866 patient separations. Of these, 96 interventions (7%) were judged to have reduced the patient's length of stay in hospital and 156 (11%) were deemed to have reduced the potential for the patient to be readmitted to hospital. The clinical significance of the intervention was deemed to be life saving in 15 (1.1%), major in 351 (25%), moderate in 535 (38%) and minor in 425 (30%).

### Evidence for systems improving information transfer

#### Information transfer at the hospital-community interface

In the previous review [2] it was noted that controlled studies undertaken in Australia to assess the impact of discharge medication management services implemented by pharmacists or by pharmacists and nurses showed improvements on patient outcomes and reductions in undesirable medication events. Further research on this type of service has been subsequently published.

A randomised, single blind, controlled trial conducted in South Australia examined whether the addition of a pharmacist transition coordinator could impact on medication management and health outcomes in older people undergoing transition from a hospital to a long-term aged care facility [46]. The study included 110 older adults discharged from three metropolitan hospitals to long-term care. The transition coordinator focused on the transfer of medicines information to care providers in the long-term care facility and the patient's family physician and community pharmacist. This included a medication transfer summary (which supplemented normal discharge information), coordination of a medication review by the pharmacist contracted to the facility and a case conference including the pharmacist coordinator, family physician, community pharmacist and registered nurse from the facility. The main outcome measure (the Medication Appropriateness Index -MAI) was assessed at discharge (baseline) and at 8 weeks post-discharge by independent pharmacists blinded to patient group allocation. The MAI was not significantly different between the groups at baseline (intervention group 3.2 [95%CI 1.8-4.6]; control group 3.7 [95% CI 2.2-5.2]), while at 8 weeks the MAI was unchanged in the intervention group (2.5 [95%CI 1.4-3.7]), but significantly higher (worse) in the control group (6.5 [95% CI 3.9-9.1]). When patients who were alive at the 8 week follow-up were included in the analysis, there were significantly fewer hospital admissions and unplanned emergency department attendances in the intervention group (RR 0.38 [95% CI 0.15-0.99]). However, there was no significant difference if all patients were included in the analysis. The intervention group reported less "worsening pain" compared to the control group (RR 0.55 [95% CI 0.32-0.94]). No difference in adverse drug events was detected (RR 1.05 [95% CI 0.66-1.68]). There were no significant differences for falls, worsening mobility, worsening behaviour or increased confusion. This study suggests a transition coordinator can improve aspects of medication management during the transition from hospital to residential aged care, however, no impact on adverse drug events was demonstrated. This study is limited by its small sample size and larger studies may be required to determine whether the endpoints are sensitive to this type of intervention.

Qualitative research has been published exploring the potential role of a liaison pharmacist between hospital and community health care settings in Australia [47]. This study involved semi-structured interviews and a focus group examining the discharge process, liaison between hospital and community settings and the possible role of a community liaison pharmacist. Participants included medical practitioners, community nurses, community pharmacists, hospital pharmacists, consumers and hospital administrators from a division of general practice in Victoria. In general, participants felt that a community liaison service should be targeted to those most a risk of medication misadventure. Potential roles for the service included providing advice and reassurance about medications, assessment of a patient's medication understanding and ability to manage their medicines at home, education and reinforcement of instructions about medicines and communication of patient progress with service providers. In general, domiciliary visits were considered the most appropriate mechanism, however telephone calls were also suggested. The logistics of providing the service included the need for domiciliary visits to be conducted within one week of discharge, but preferably within 24-48 hours.

Other models for development of medication liaison services between hospital and community settings have been examined in major hospitals in NSW and South Australia.

In NSW a 'Heartlink' medication management pathway for patients with chronic heart failure was developed involving a community liaison pharmacist and medication management review facilitator [48]. On hospital admission, patient consent was obtained for the hospital pharmacist to communicate with the patient's preferred community pharmacy to obtain a complete medication history. The community pharmacist inserted an alert onto the patient's computer file. On discharge from hospital the patient was given a medication list by the hospital pharmacist, and the community pharmacist was sent the discharge prescription, information about medication changes, any relevant information to assist long-term patient monitoring and risk factors for medication misadventure. A community liaison pharmacist requested the patient's GP refer the patient for a home medication review (HMR) and provided the GP with information about specific medication risk factors or recommendations from the hospital team. The liaison pharmacist accompanied the accredited pharmacist on the HMR visit to provide written resources and other information to the patient. An HMR facilitator worked to provide information to GPs and community pharmacists about the service. A retrospective survey of GPs, community pharmacists and accredited pharmacists found most agreed that the service improved the link between the hospital and community setting. Time factors and lack of patient interest was identified as the major barriers to the HMR process. Patients receiving the service who responded to the questionnaire (n = 27) reported they were more confident taking medications regularly after the HMR and felt they learnt something from the HMR (24 respondents, 89%).

In South Australia a pilot study examined a service from within the hospital (before discharge) to organize an HMR for patients at high risk of medication misadventure [49]. Standard care involved mailing a discharge summary to the patient's GP. The added service involved a liaison pharmacist:

- sending a medication discharge summary to the patient's GP and community pharmacist;

- organising an appointment for the patient with their GP two days after discharge to order a HMR;

- requesting the community pharmacist arrange an accredited pharmacist to undertake the HMR or ordering a hospital-funded review if the GP and community pharmacist could not be involved;

- sending the HMR report to the hospital outpatient clinic, the GP and community pharmacist.

Of the 50 eligible patients, 38 gave consent and 21 patients received the full service. The mean time for the HMR was 18 ± 7 days post-discharge. Barriers to the service included GP time constraints and unwillingness to learn how to make an HMR referral. Community pharmacist barriers included time constraints and a lack of remuneration.

#### Information transfer between hospitals and general practitioners

An Australian study to promote medication information transfer at the hospital community interface focused on the transfer of information between general practitioners and hospitals at both hospital admission and discharge [50]. This quasi-pre-test post-test design study aimed to improve communication between general practitioners and hospital staff in an Area Health Service in NSW. Stage one of the project [51], indicated that compliance with the Australian Pharmaceutical Advisory Council (APAC) *National guidelines to achieve the continuum of quality use of medicines *between hospital and community was poor, and that a number of barriers to effective communication existed. A series of workshops were conducted which identified changes that could be made to overcome these communication barriers. In stage two, progress was assessed using specific indicators. GPs (n = 122) completed questionnaires for two of their elderly patients discharged from a hospital in the area health service. Subsequently, a forum was held to review results and reassess action plans. Three months later another survey was conducted. In comparison with stage one, there were substantial and maintained improvements in faxing of discharge summaries from hospitals to GPs (p < 0.001) and provision of medication information to hospitals by GPs for patients at risk (p < 0.05). Some problems, however, had changed little including a poor rate of hospital notification to GPs of a patient's admission to hospital. This study did not use adverse drug events or medication error as an outcome measure.

#### Shared electronic medication records

Initiatives to develop systems to improve the sharing of medication information between patients and various healthcare providers through a shared electronic medical record have been funded through the Australian Government. Since the last medication safety review [2] a MediConnect program (formerly the Better Medication Management System) began development. This program aimed to develop a system to allow consumers to consent to various different healthcare professionals accessing and, where necessary, using and recording information in a shared medication record. A secure national electronic system was trialled successfully in two sites in Victoria and Tasmania in 2003. In 2004 the MediConnect program was incorporated within the wider HealthConnect program [52]. HealthConnect is an ongoing partnership between the Commonwealth and State and Territory Governments to facilitate sharing of health information electronically. Systems for electronic sharing of patient health information, including medication information between different settings are currently under development and evaluation in various states [53].

#### Medication record cards

The previous medication safety review [2] identified one randomised controlled study in the hospital outpatient setting which assessed the impact of using a medication card in conjunction with medication counselling for improving knowledge about medications and compliance [54,55]. No further Australian studies were located.

### Evidence for systems promoting multidisciplinary care

#### Medication Management Review and Case Conferencing Services

The only controlled Australian studies assessing medication management and case conferencing services have been undertaken as part of hospital-community discharge liaison studies [46,56-59]. No further studies in the acute care setting in Australia were located.

### Evidence for systems to promote reporting of medication incidents and adverse drug reactions

The previous medication safety review did not include analysis of the evidence for improving adverse drug reaction reporting or incident reporting [2], however it highlighted that routine data collection about undesirable medication events is important for understanding why medication incidents occur and how they might be prevented. While there are well established mechanisms to collect data on adverse drug reactions and medication incidents in Australia, medication incidents and adverse drug reactions are still under-recognised and under-reported. Continued and increased participation needs to be encouraged. Some strategies described in the recent literature have been developed to promote participation in reporting.

A strategy to increase the reporting of adverse drug events in the Alfred Hospital in Melbourne involved ensuring medication errors identified via calls for the medical emergency team (MET) were included in the hospital quality programs [60]. The MET provides early intervention when a patient's condition deteriorates and causes of the deterioration are recorded. A modification of the MET data management process involved including a category on the reporting form for "adverse medication effect" and a process to ensure that reports where medications were a contributing factor were communicated monthly to the pharmacy department. This allowed review and inclusion in the hospital continuous quality improvement program. The intervention enabled detection of adverse drug events that would have been missed in the existing system.

Other studies have examined factors that influence adverse drug reaction (ADR) reporting [61] and incident reporting [62] in the acute care setting in Australia. Suggested strategies to improve reporting included improving the accessibility of report forms, encouraging computer-based reporting and implementing educational initiatives for nurses and junior medical staff [61]. In response to barriers to incident reporting [62] suggestions included development of time-efficient reporting systems and resources to provide feedback and action in relation to reported incidents and near-misses. Personal digital assistants (PDAs) were successfully utilized by anaesthetists to facilitate incident reporting [63]. There is a need for further research in the Australian acute care setting to evaluate whether these suggested strategies could increase reporting rates.

### Evidence for systems-based approaches to understanding and preventing medication errors

#### Systems to allow health services to assess medication systems and performance

The National Medication Safety Breakthrough Collaborative was a key initiative of the former Australian Council for Safety and Quality, which aimed to reduce harm from medications. The collaborative of 100 health service teams worked towards a goal of reducing medication-related harm to patients by 50% [64]. Reported achievements included more than halving the percentage of patients experiencing a high-risk adverse drug event (by the top 8 hospital teams) and an increase in the percentage of hospitalised patients who had medicines information communicated to their primary health care providers in a timely manner - from less than 30% to over 90% (by the top 8 hospital teams) [64]. The collaborative developed "toolkits" to improve medication safety, including alert cards, incident report forms, education tools for staff and patients, communication tools and guidelines for high-risk medications [65,66].

The NSW Therapeutic Advisory Group (NSW TAG) and the Clinical Excellence Commission have adapted resources developed by the North American Institute for Safe Medication Practices (ISMP). The "*Medication Safety Self Assessment for Australian Hospitals*" and "*Medication Safety Self Assessment for Antithrombotic Therapy in Australian Hospitals*" are available through the NSW Government Clinical Excellence Commission (CEC) website [67]. Completed assessments can be submitted to the CEC through a secure site which provides a confidential online report back to the hospital. The resources are designed to allow hospital administrators, in conjunction with a multidisciplinary team, to self assess their hospital's performance on elements that have been shown to improve safe use of medicines generally and anti-thrombotic agents specifically. The system is currently used, but has not been evaluated for its impact on improving medication safety [68].

#### System-based approaches to drug administration errors

A study conducted at a NSW tertiary hospital examined a systems-based approach to reporting, review and feedback of data obtained on prescribing incidents in the hospital [69]. A database of prevented prescribing incidents (near-miss incidents) detected by hospital pharmacists was developed. A pharmacist classified incidents by type and potential severity, and recorded descriptive data and a brief narrative of the incident. Systems failures were identified from the data and fedback to specific clinical areas and specialist medical officers. Clinical pharmacists specialising in the clinical area were involved in multidisciplinary forums with junior and senior medical officers in which intervention reports were discussed. A survey was sent to 21 senior clinicians who received reports through the program, of which 10 (47%) responded. All indicated that the feedback was of value in improving prescribing practice and was incorporated into clinical quality programs. Most respondents (80%) indicated they found the comparative data between departments useful.

A program to reduce the potential for medication infusion-related error was undertaken at an acute care hospital in Melbourne [70]. A multidisciplinary team examined medication administration errors over a 29-month period identifying root causes and contributing systems failures. Identified systems failures included design flaws in technology currently in use, deviations from safe practice that had become culturally accepted, complex and variable medication prescribing, unnecessary administration practices, lack of accessible medication calculation resources and limited accessible drug information. Improvement initiatives included a medication safety education program incorporating medication calculations initiatives, a campaign to increase reporting of near-miss incidents, strategies to address unsafe practices that had become accepted in the hospital such as storing potassium chloride ampoules in bedside drawers with other medications and poor labelling of drug infusions. Other initiatives included the implementation of an auditing program, changes to infusion pump equipment and methods to standardize the prescribing and administration of particular medications. The impact of the program on medication infusion error rates in the hospital was not reported.

These studies describe initiatives to design system-based approaches to reduce drug administration errors. There is a need for further research in the Australian acute care setting examining the actual impact of these approaches on medication errors and adverse drug events.

## Conclusion

In 2002, the former Australian Council for Safety and Quality in Health Care, in its national report on medication safety, highlighted a number of systems solutions known to be effective in improving medication safety [2]. These included clinical decision support systems; adverse drug event alerts; systems that provide adequate checking, such as bar coding; individual patient medication supply systems; as well as provision of clinical pharmacy services and discharge medication management services. Since this review, further Australian studies addressing the evidence base and experiences in their implementation have now been published. However, gaps still exist.

There has been significant progress in strategies to improve prescription writing for medications in hospitals with the introduction of a NIMC now used in all Australian public hospitals and many private hospitals. Published research has highlighted limitations with the chart, however there are systems in place to allow ongoing evaluation and nationally coordinated strategies for making changes to the chart design.

Studies have now assessed the implementation of computerised prescribing and clinical decision support in Australian hospitals, suggesting computerised prescribing alone without decision support, may lead to increased error. Studies have also highlighted that implementation of these systems in acute care must include appropriate education and training for staff, a change management strategy and a highly organised approach at all levels of the institution, giving consideration to the technical issues and culture and environment in which it is to be used. Similarly, the need for standardisation of systems and an agreed set of national standards has been highlighted. There remains a lack of published research on the impact of electronic prescribing in combination with CDSS on medication errors or adverse drug events when used by health care practitioners in the acute care setting in Australia.

New strategies that have been assessed include double checking versus single checking by nurses for safe medication administration and patient self-administration in hospital. The small studies found no significant differences between groups, however, the studies were only located in single centres and possibly had insufficient power to detect differences. While these studies do not provide sound evidence of effectiveness, they warrant further research in these areas.

Other strategies that have been implemented but the impact on medication error rates not reported include leur incompatible systems to avoid incorrect route of administration for intravenous and intrathecal injections, as well as the removal of concentrated potassium chloride from wards with replacement by pre-mixed solutions.

Academic detailing has been demonstrated to reduce errors in prescriptions for Schedule 8 medicines where error rates were high and an uncontrolled study suggested an education program was effective in reducing the use of error prone prescribing abbreviations in the emergency department setting.

Studies have continued to assess hospital discharge planning or liaison pharmacy services primarily focusing on implementation issues. Barriers to home medication reviews after hospital discharge include workload factors for both general practitioners and pharmacists and lack of patient interest, as well as the ability to engage an accredited pharmacist in a timely manner. One new model included a transition co-ordinator to assist transfer of medication information for patients discharged from hospital to residential aged-care facilities. The model demonstrated an improvement in medication appropriateness, however there was no significant impact on adverse drug events, but the study may have had insufficient power to detect this endpoint.

No new studies were located that have assessed the impact of individual patient medication supply, adverse drug event alerts or bar coding. The extent of their implementation across Australia is unknown.

While there is now a stronger evidence base demonstrating that systems factors are major contributors to medication errors, there is still very limited research assessing the impact of an integrated set of activities on medication safety.

## List of abbreviations used

ADR: (adverse drug reaction); CDSS: (clinical decision support systems); CPOE: (computerised physician order entry); DOA: (drugs of addiction); ED: (emergency department); MAI: (medication appropriateness index); NIMC: (National Inpatient Medication Chart); NSW: (New South Wales); RN: (registered nurse).

## Competing interests

The authors declare that they have no competing interests.

## Authors' contributions

SJS was the main author of Part 2 of this review and was involved in reviewing the literature, summarising study findings and synthesis of the findings with those from the previous medication safety review. EER made a substantial contribution to drafting and editing of this paper and provided direction and guidance in the review of the relevant literature.

## Authors' informations

EER is an Associate Professor and co-director in the Quality Use of Medicines and Pharmacy Research Centre (QUMPRC), Sansom Institute, University of South Australia. SJS is a Research Fellow in the QUMPRC. EER and SJS were the primary authors of the *Second National Report on Patient Safety Report - Improving Medication Safety *for theAustralian Council for Safety and Quality in Health Care in 2002.
